# Hospital resuscitation teams: a review of the risks to the healthcare worker

**DOI:** 10.1186/s40560-017-0253-9

**Published:** 2017-10-11

**Authors:** Stephen M. Vindigni, Juan N. Lessing, David J. Carlbom

**Affiliations:** 1 Division of Gastroenterology, Department of Medicine, University of Washington, 1959 NE Pacific Street, Box 356424, Seattle, WA 98195-6424 USA; 2 Division of General Internal Medicine, Department of Medicine, University of Colorado, 13001 E 17th Place, Aurora, CO 80045 USA; 3 Division of Pulmonary, Critical Care and Sleep Medicine, Department of Medicine, University of Washington, 1959 NE Pacific Street, Seattle, WA 98195-6424 USA

**Keywords:** Code blue, Code team, Cardiopulmonary resuscitation, CPR, Advanced cardiac life support, Hospital rapid response teams, Medical emergency teams, Occupational medicine

## Abstract

**Background:**

“Code blue” events and related resuscitation efforts involve multidisciplinary bedside teams that implement specialized interventions aimed at patient revival. Activities include performing effective chest compressions, assessing and restoring a perfusing cardiac rhythm, stabilizing the airway, and treating the underlying cause of the arrest. While the existing critical care literature has appropriately focused on the patient, there has been a dearth of information discussing the various stresses to the healthcare team. This review summarizes the available literature regarding occupational risks to medical emergency teams, characterizes these risks, offers preventive strategies to healthcare workers, and highlights further research needs.

**Methods:**

We performed a literature search of PubMed for English articles of all types (randomized controlled trials, case-control and cohort studies, case reports and series, editorials and commentaries) through September 22, 2016, discussing potential occupational hazards during resuscitation scenarios. Of the 6266 articles reviewed, 73 relevant articles were included.

**Results:**

The literature search identified six potential occupational risk categories to members of the resuscitation team—infectious, electrical, musculoskeletal, chemical, irradiative, and psychological. Retrieved articles were reviewed in detail by the authors.

**Conclusion:**

Overall, we found there is limited evidence detailing the risks to healthcare workers performing resuscitation. We identify these risks and offer potential solutions. There are clearly numerous opportunities for further study in this field.

## Background

“Code blue” events are cardiopulmonary resuscitation efforts that occur in hospital settings. A dedicated multidisciplinary resuscitation team rapidly convenes at the bedside, initiates cardiopulmonary resuscitation (CPR), and performs an assessment of the situation. Cardiac defibrillation, establishment of intravenous (IV) access, placement of an advanced airway, blood draws, and medication administration, among other tasks, are integrated into these code situations. Multiple hospital staff are frequently present including physicians at all training levels, medical students, nurses, critical care staff, laboratory technicians, social workers, and clergy; increasingly, patient family members are also at the bedside.

The primary goal of resuscitation efforts is revival of the patient, and there is extensive literature discussing the management of cardiopulmonary arrest. There is, however, a dearth of literature commenting on the risks to the code team performing the resuscitation. One author experienced severe neck pain diagnosed as an epidural cervical hematoma following multiple rounds of CPR during a code. This event prompted a literature search to see if other providers experienced similar ill health effects, but we were able only to identify one editorial in the nursing literature that broadly discussed code-related occupational hazards [1]. Thus began our review of the available literature, which revealed many risk categories, which include infectious, electrical, musculoskeletal, chemical, irradiative, and psychological components (Table 1).

**Table 1:** Risks to in-hospital resuscitation teams and potential preventive strategies to mitigate risk

Risk category	Specific risks and potential exposures	Potential preventive actions and solutions
Infectious	□ Percutaneous/needlestick injuries □ Respiratory/airborne exposures □ Contact exposures □ Emerging/re-emerging infections	□ Convenient sharps disposal □ Use of needles with safety features □ Blood-borne pathogens training for all employees □ Reporting of needlestick injuries with post-exposure medical evaluations and prophylaxis □ Breathing filter during mask ventilation □ Clearly defined roles for staff regarding: who is responsible for blood draw, central line placement, etc.
Electrical	□ Shock during defibrillation □ ICD misfiring □ Fire generation near oxygen-rich atmospheres	□ Standard maintenance of defibrillators □ Training of resuscitation team members on the use of defibrillators □ Placing a donut magnet over ICDs; consider including on code cart □ Clear announcement of impending defibrillation □ Preferential use of gel adhesive pads instead of hand-held paddles. If paddles are used, avoidance of excess amounts of conduction gel □ Consider removal of supplemental oxygen from bed prior to defibrillation
Musculoskeletal	□ Neck/back injuries during/following chest compressions □ Falls while running to code situations	□ Training to providers on proper posture and chest compression technique □ Adjust height of bed during chest compressions and/or use of step stools □ Adequate number of chest compressors to allow recovery and reduce resuscitator fatigue
Chemical	□ Risks of chemical warfare	□ Programs for decontamination of victims of chemical warfare
Irradiative	□ Exposure during cross-table cervical spine radiographs with manual cervical spine stabilization, generally in trauma patients □ Brachytherapy patients	□ Maximize distance between provider and radiation beam □ Use of lead-lined gloves, lead aprons, thyroid shields, and glasses
Psychological	□ Traumatic stress with short- and long-term mental and physical impact	□ Stress management programs □ De-briefing following resuscitation efforts, ideally within less than 72 h and in a non-threatening manner □ Counseling and related programs for depression, PTSD and, overall mental health well-being □ Implementation of “death rounds”

Through better understanding of the potential harms to resuscitation teams, we have the opportunity to mitigate or prevent them. The aim of this review is to summarize the available literature regarding occupational risks to medical emergency teams, characterize these risks, offer preventive strategies, and highlight the need for further research.

## Materials and methods

**Fig. 1:**
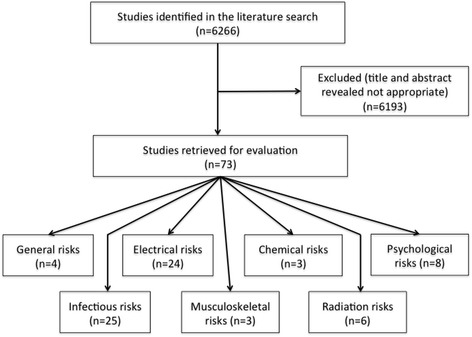
Study selection algorithm

We performed a review of peer-reviewed publications with a broad systematic literature search using PubMed to identify articles that discuss potential occupational hazards during resuscitation scenarios. Only articles published in English were reviewed. PubMed was searched for all historical articles through September 22, 2016. A medical librarian assisted with developing the literature search strategy. All identified articles were reviewed by two authors (SV, JL) with relevant information abstracted. Additional articles were identified from the reference sections. Based on review of the articles found, six risk areas were identified. Using this information, the search strategy was further refined to use the following keywords: “occupational exposure” or “code blue” or “resuscitation” or “trauma team” or “cardiopulmonary resuscitation” or “CPR” and “electric” or “chemical” or “musculo” or “musculoskeletal” or “psych” or “mental” or “infectious” or “infection” or “radiation.” We reviewed all study types including randomized controlled trials, cohort and case-control studies, reviews, case reports and case series, and editorials. In total, 6266 studies were identified in the literature with 73 meeting the criteria to be included and reviewed across six categories (Fig. 1).

## Results and discussion

### Infectious risks

Multiple studies have discussed the benefits of universal precautions in healthcare settings, including during code situations. While focus has traditionally been placed on viral hepatitis (e.g., hepatitis B and C) and human immunodeficiency virus (HIV), providers are at risk for exposure to multiple infectious agents via cutaneous, mucosal, and percutaneous routes. In the late 1980s and 1990s, significant focus was on the risk of HIV exposure with multiple studies making strong arguments for universal precautions during code situations [2].

While the incidence of needlestick injuries among healthcare workers during routine medical care has been well documented in the literature, code situations undoubtedly present a higher risk. During resuscitative attempts, multiple providers are using needles for venipuncture, arterial blood gas sampling, and emergent placement of central venous catheters, often during active patient motion related to repositioning or CPR efforts. Despite this, very little has been published on the topic of needlestick injuries during resuscitation. One case report describes a resident physician who sustained a needlestick injury while attempting to place a central line; he appropriately reported the injury and received post-exposure prophylaxis [3]. But outside of this case report, no additional data was found. While not a needle exposure, there is one case report of a critical care nurse who sustained a puncture wound during chest compressions through contact with a patient’s sternotomy wires following prior cardiac surgery; there were no reported infectious complications [4].

Given the perceived increased risk of parenteral exposure, measures beyond universal precautions are essential, including the use of safer syringes using newer engineered controls. These devices have a mechanism that retracts or covers the exposed needle to prevent accidental exposure following patient intervention [5, 6].

There is also the increasing use of intraosseous (IO) catheters leading the American Heart Association (AHA) to endorse IO cannulation as an appropriate means of access during resuscitation in the patient without available IV access [7]. While there are no documented adverse events to resuscitation teams, we anticipate these devices are safer and likely pose less risk to healthcare providers when compared to emergent femoral or other central line access placement.

Another area for potential infectious risk is respiratory exposure. While earlier resuscitation efforts included mouth-to-mouth resuscitation—with very rare documentation of infectious agent transmission—newer guidelines focus on chest compressions and no longer include direct mouth-to-mouth contact without the use of a protective barrier [8–10]. Historical concerns included a multitude of oral-to-oral infectious agents, including tuberculosis, HIV, herpes simplex, and *Helicobacter pylori*; these risks are no longer present [11]. In hospital-based settings, ventilation is performed through a bag-valve mask. Mask ventilation may contribute to the spread of infection exposing chest compressors to infectious air particles, but this is uncommon; a breathing filter may eliminate this risk [12]. The act of intubation and suctioning prior to intubation likely increases the risk of airborne or respiratory exposure, as seen during the severe acute respiratory syndrome (SARS) outbreak of 2003, but following intubation, manual ventilation and suctioning did not significantly increase risk [13]. Despite this, there is still at least a low-level risk present as providers are in contact with patient secretions.

Contact transmission is also rare. Theoretically, there may be increased exposure in patients with methicillin-resistant *Staphylococcus aureus* or vancomycin-resistant *enterococci*, but if universal precautions are implemented with gloves (and ideally gowns), this risk is reduced. First responders in the field may be at greater risk given the lack of a controlled setting with one case report describing a firefighter exposed to a child’s oral secretions leading to *Streptococcus pyogenes* cellulitis at the site of an abrasion [8, 14]. There are no documented inpatient complications during resuscitation efforts.

Finally, there is always concern for emerging or re-emerging infections. Recent examples have been avian influenza, SARS, and Ebola virus disease [15–17]. These events are rare and unpredictable. There is one case report describing exposure to the H1N1 influenza virus when an endotracheal tube leaked during open-chest cardiac massage resulting in aerosolization of the virus [15]. Ulrich and Grady also describe the ethical concerns around cardiopulmonary resuscitation in Ebola patients and stress the importance of personal protective equipment and training of healthcare workers [16]. They also raise the difficult question of futility of CPR in Ebola-affected patients, particularly in the setting of higher risks to rescuers; they acknowledge Ebola can present with a spectrum of symptoms and some patients may benefit from CPR more than others.

In general, infections tend to be at the forefront of most providers’ minds when they think about the risks related to resuscitation efforts. Despite this, true documented transmission is rare with percutaneous injury the most concerning and should be even less of a risk with accessible sharp containers and the aforementioned use of safer, advanced syringe devices. Additionally, there should be clearly assigned roles for who will be performing procedures involving sharps so as not to create confusion and ensuring only trained healthcare workers are performing needle-based interventions.

### Electrical risks

Although significantly less common than a needlestick injury, there is a risk of electrical injury to code teams, particularly during defibrillatory shocks administered for treatment of ventricular fibrillation and pulseless ventricular tachycardia rhythms. These risks are particularly important as access to defibrillators by bystanders and non-healthcare providers in community settings (e.g., department stores, airports, etc.) has become increasingly common. A systematic review identified 29 adverse events during defibrillator use [18]. Excluding intentional or misuse of defibrillators (e.g., attempted suicide), three incidents were associated with faulty equipment (e.g., crack in paddles, inappropriate discharge) and four occurred during training or maintenance of equipment (e.g., accidental discharge). Fifteen accidental shocks during resuscitation could not be attributed to faulty equipment. Most were due to healthcare workers coming into contact with the patient or the stretcher, with few cases attributed to arc discharge between paddles and the patient’s chest. The most common adverse effects were burns and tingling sensations [19]. The use of adhesive gel pads has limited the need for hand-held paddles, which has further reduced the risk of inadvertent shock [20].

Many studies have stressed the importance of limiting interruptions of chest compressions to reduce falls in coronary and cerebral perfusion pressure. To accomplish this, charging the defibrillator while compressions are ongoing is now recommended [21, 22]. Eliminating this delay ensures greater compression fraction (i.e., the percentage of time during which chest compressions are being delivered) but may also increase the risk of contact with the patient during defibrillation. Edelson et al. performed a multi-center, retrospective study of defibrillator charging by analyzing CPR-sensing defibrillator transcripts for pre-shock pauses and total hands-off time [21]. With charging during CPR, hands-off time was decreased and only one shock was administered with chest compressions ongoing; the compressor was unaffected. The data supports the AHA recommendation that defibrillator charging during chest compressions is safe [23].

More recently, there has been the suggestion of maintaining chest compressions during defibrillation with the hypothesis that the shock risk is low if gloves are worn by providers [24, 25]. Studies analyzing the electrical resistance of nitrile gloves used during codes compared to unused, control gloves found that gloves became degraded during wear and especially during active chest compressions (e.g., microscopic tearing, conductive moisture). There was a decrease in resistive protection; therefore, gloves were considered inadequate electrical insulation for ongoing contact with the patient during defibrillation [26]. Additional studies have shown similar results with vinyl and nitrile gloves [27, 28]. Lemkin asserts that the leakage current does not determine the risk of defibrillation, particularly since the amount of energy transferred is dependent on total energy delivered, voltage, and resistance of the patient [29]. Using cadavers to map rescuers’ voltage exposure during defibrillation, he concluded hands-on defibrillation poses a risk to chest compressors without a clear negative impact of lifting hands for < 5 s on patients. The study results are debated as an overestimation of risk, but currently active hands-on defibrillation cannot be endorsed, and brief compression pauses are still recommended during shock delivery [30, 31]. The development of a “resuscitation blanket”—a layer between the patient’s chest and rescuers’ hands to prevent shock exposure—has been proposed but has not been incorporated into practice [32].

An additional potential electrical exposure is the firing of implantable cardioverter defibrillators (ICDs) during resuscitation. Clements presents a case report of a 75-year old man with pulseless electrical activity undergoing CPR [33]. The ICD was found to deliver four shocks during CPR that had no effect on the resuscitators; however, one shock was delivered during cardiac massage resulting in a shock to the massager and an inability to return to work for at least 30 min. The cardiac massage was hypothesized to mimic a shockable rhythm. An additional case report by Siniorakis describes a chest compressor who received an ICD-related shock that threw him against a wall resulting in neck and back pain [34]. The “electrical noise” generated by chest compressions was believed to have been interpreted by the ICD as ventricular fibrillation. Other case reports describe ICD-related paresthesias [35]. The current guidance is placement of a donut magnet over the ICD to eliminate the risk of ICD firing.

Despite the above risks, the incidence of significant shocks is low, and while defibrillation is considered a risk to healthcare workers, the fear of significant shock injury (as often inaccurately depicted by Hollywood) is unwarranted.

An additional rare electrical risk to note is fire related to defibrillation performed near flammable material, such as oxygen [18, 36]. Historical reports suggested removal of oxygen masks during defibrillation; however, more recent recommendations assert the low risk of fire is outweighed by the risks of delayed defibrillation and possible endotracheal dislodgement [37–39].

### Musculoskeletal risks

The act of performing chest compressions is a strenuous task for even the fittest of healthcare workers. There are multiple potential injuries that can be sustained from shoulder to neck to back injuries. Musculoskeletal strain may not be apparent at first, tempered by the rush of adrenaline during a code situation but may become more evident in the days following a resuscitation effort.

There is a dearth of published literature discussing the musculoskeletal impact of CPR on the resuscitator. Cheung et al. performed a prospective, observational, interview-based study of medical emergency teams to assess physical injuries during hospital emergencies [40]. Injuries included back or shoulder pain following chest compressions, slipping en route to a resuscitation code, and exposure to urine, feces, blood, or vomitus. Of 17 injuries recorded, only one required treatment and time off from work. The injury rate was 13 per 1000 emergency team participants. Based on these results, the risk of injury was overall low and injuries that did occur were usually minor and without short- or long-term effect on daily activities [40].

Jackson and Sturrock describe a case of “resuscitation shoulder” or the partial tear of a rotator cuff experienced by a resident physician after performing repetitive and prolonged chest compressions on several patients over three consecutive nights while on call [41]. Similar injuries have been described following repetitive athletic pursuits.

Anecdotally, these authors are aware of a resident who developed an anterior cruciate ligament tear following a fall while running to a code. As mentioned above, one author developed acute neck pain following multiple rounds of vigorous CPR during an overnight code. He ultimately developed neurological symptoms with tingling in his fingers and was found to have a cervical epidural hematoma that was managed conservatively with improvement in symptoms. There is also a news media story of a paramedic developing a myocardial infarction (MI) while performing CPR on a patient experiencing an MI [42].

The effect of rescuer fatigue during prolonged codes has also been discussed with decreased compression depth achieved [43]. It can be hypothesized that as resuscitators develop fatigue, they may develop altered posture and increase the risk of musculoskeletal strain or sprain.

Additional factors to consider include space availability as limited by room size and number of providers in the room, bed type (emergency stretcher vs. standard hospital bed), height of the bed and availability of step stools for shorter providers, length of the code, improper rescuer positioning, and patient characteristics (e.g., obesity). These factors may predispose and/or increase the risk of injury during a resuscitation. In addition to prevention of musculoskeletal strain, a recent simulation study found the use of a step stool (23 cm in height) was associated with improved compression depth [44].

### Chemical risks

We did not identify any literature focused on chemical risks during in-hospital resuscitation. There are a few publications commenting on the risk of chemical exposure to rescuers in mass causality or chemical warfare scenarios, but not in hospital settings. Depending on the chemical agent, patients may be at higher risk of cardiopulmonary arrest. Since healthcare providers have the potential to be exposed to victims of chemical warfare, protocols should be established to prepare for decontamination of these patients and ensure the protection of healthcare workers [45]. Interestingly, a study on the performance of paramedics wearing chemical protective suits found impairment of fine motor skills (e.g., IV cannulation, subcutaneous epinephrine injection) but overall successful resuscitation (e.g., defibrillation, tracheal intubation), despite delays imparted by wearing the suit [46]. This scenario may be translated to inpatient codes in the rare event of a chemical outbreak.

### Radiation risks

Radiation risk is equally uncommon during resuscitation efforts. A rare exception is for patients who arrest during a radiographic examination, although the imaging study would be terminated in this setting. Most publications commenting on radiation risk relate to trauma patients who present in the emergency department and are being stabilized, which is a different scenario than a true cardiopulmonary arrest. In these trauma situations, there is possible radiation exposure to healthcare workers if manual cervical spine stabilization is required and cervical spine radiographs are taken, but more often, the patient is stabilized and images are obtained quickly with healthcare staff in a protected area and often wearing leaded protective equipment. Lead aprons, leaded gloves, thyroid shields, and glasses should be available for use during radiographic studies of trauma patients in these rare situations [47].

An additional potential radiation exposure is when resuscitation efforts are required in patients with implanted radioactive sources, such as in brachytherapy, for treatment of various cancers. For these patients undergoing surgery, Basran et al. suggest the use of lead-based gloves and use of dosimetry to measure exposure [48]. In known patients with active radioactive sources who code, these protective approaches may also apply.

### Psychological risks

Resuscitation efforts, even if successful, may have a dramatic psychological impact on resuscitation team members. While the greatest literature on the mental health impact of rescuers is related to disasters and mass tragedy, such as following the 9/11 response, there is less evidence on the psychological impact of in-hospital resuscitations. As resuscitations are often unexpected, it may be difficult for healthcare workers to adapt and there is a risk of personal crisis and traumatic stress [49]. Stress can also have physical effects, including headache, chronic pain, and hypertension with the potential for absenteeism, impaired decision-making, and effects both at work and home [49, 50].

With the premise that nurses may experience long-term stress effects following a resuscitation, Cudmore performed a survey of nurses exploring the perceived need for debriefing following the resuscitation of a patient [51]. Nurses supported a formal debriefing session, particularly if the resuscitation was difficult or upsetting, such as involving a child, more than one patient, or related to major trauma or burns. Gamble discussed a framework for debriefing including introduction of resuscitation team members, discussion of case facts, an emotional description of the events and nurse response, identification of learning opportunities, and summing up a plan of action [52]. Based on our review, there are no studies focused on physician response to stress following resuscitation.

While not immediately related to resuscitation efforts, some institutions have implemented “death rounds” to discuss the emotions surrounding a patient death. While generally focused in a palliative care setting more commonly than a code blue scenario, the presence of a supportive environment to discuss difficult situations is likely to be beneficial [53–55].

## Limitations

The greatest limitation (and significant finding) of this review is a lack of published literature on this topic. Of existing literature, the quality and rigor is variable as numerous cited studies are case reports rather than higher levels of evidence, such as randomized controlled trials or meta-analyses. While our literature review was thorough, it is possible additional risk categories exist. Given that medical errors and description of workplace injuries are often underrepresented in the literature, we anticipate that there are significantly more episodes of resuscitation harms that have not been documented nor published, especially the psychological impact to providers imparted by these stressful situations.

## Conclusions

As the population ages, inpatient medical teams will continue to be engaged in resuscitation scenarios, possibly with increased frequency. This orchestrated resuscitation possesses inherent risks for the providers that include infectious, electrical, musculoskeletal, chemical, irradiative, and mental health threats. For each of these, strategies can be taken to reduce, if not prevent, risk. In addition to identifying these risks and potential preventive approaches, this review also highlights the overall lack of evidence of this topic area. While the patient is appropriately the focus during resuscitation efforts, we must not neglect the providers who need to remain in good health for the next code blue echoed over the loud speaker.

## Abbreviations

AHA: American Heart Association
CPR: Cardiopulmonary resuscitation
HIV: Human immunodeficiency virus
ICDs: Implantable cardioverter defibrillators
IO: Intraosseous
IV: Intravenous
MI: Myocardial infarction
SARS: Severe acute respiratory syndrome
